# Occupational Exposure to Ultraviolet Radiation and Risk of Non-Melanoma Skin Cancer in a Multinational European Study

**DOI:** 10.1371/journal.pone.0062359

**Published:** 2013-04-24

**Authors:** Simona Surdu, Edward F. Fitzgerald, Michael S. Bloom, Francis P. Boscoe, David O. Carpenter, Richard F. Haase, Eugen Gurzau, Peter Rudnai, Kvetoslava Koppova, Joëlle Févotte, Giovanni Leonardi, Marie Vahter, Walter Goessler, Rajiv Kumar, Tony Fletcher

**Affiliations:** 1 Department of Environmental Health Sciences, School of Public Health, University at Albany, State University of New York, Rensselaer, New York, United States of America; 2 Department of Epidemiology and Biostatistics, School of Public Health, University at Albany, State University of New York, Rensselaer, New York, United States of America; 3 Institute for Health and the Environment, University at Albany, Rensselaer, New York, United States of America; 4 New York State Cancer Registry, New York State Department of Health, Albany, New York, United States of America; 5 Department of Educational and Counseling Psychology, School of Education, University at Albany, State University of New York, Albany, New York, United States of America; 6 Health Department, Environmental Health Center, Babes Bolyai University, Cluj-Napoca, Romania; 7 Department of Environmental Epidemiology, National Institute of Environmental Health, Budapest, Hungary; 8 Department of Environmental Health, Regional Authority of Public Health, Banska Bystrica, Slovakia; 9 UMRESTTE, Department of Epidemiological Research and Survey in Transport, Work and Environment, University of Lyon, Lyon, France; 10 Department of Social and Environmental Health Research, Public Health, London School of Hygiene and Tropical Medicine, London, United Kingdom; 11 Centre for Radiation, Chemical and Environmental Hazards, Health Protection Agency, Chilton, United Kingdom; 12 Institute of Environmental Medicine, Karolinska Institutet, Stockholm, Sweden; 13 Institut für Chemie-Analytische Chemie, Karl-Franzens-Universität, Graz, Austria; 14 Division of Molecular Genetic Epidemiology, German Cancer Research Center, Heidelberg, Germany; Ohio State University Medical Center, United States of America

## Abstract

**Background:**

Studies suggest that ambient sunlight plays an important role in the pathogenesis of non-melanoma skin cancers (NMSC). However, there is ongoing controversy regarding the relevance of occupational exposure to natural and artificial ultraviolet radiation (UV) radiation.

**Objectives:**

We investigated potential associations between natural and artificial UV radiation exposure at work with NMSC in a case-control study conducted in Hungary, Romania, and Slovakia.

**Methods:**

Occupational exposures were classified by expert assessment for 527 controls and 618 NMSC cases (515 basal cell carcinoma, BCC). Covariate information was collected via interview and multiple logistic regression models were used to assess associations between UV exposure and NMSC.

**Results:**

Lifetime prevalence of occupational exposure in the participants was 13% for natural UV radiation and 7% for artificial UV radiation. Significant negative associations between occupational exposure to natural UV radiation and NMSC were detected for all who had ever been exposed (odds ratio (OR) 0.47, 95% confidence interval (CI) 0.27–0.80); similar results were detected using a semi-quantitative metric of cumulative exposure. The effects were modified by skin complexion, with significantly decreased risks of BCC among participants with light skin complexion. No associations were observed in relation to occupational artificial UV radiation exposure.

**Conclusions:**

The protective effect of occupational exposure to natural UV radiation was unexpected, but limited to light-skinned people, suggesting adequate sun-protection behaviors. Further investigations focusing on variations in the individual genetic susceptibility and potential interactions with environmental and other relevant factors are planned.

## Introduction

Non-melanoma skin cancers (NMSC) comprise more than one-third of all cancers and are increasing worldwide, causing a significant economic burden at the individual and community levels [1], [2]. The most common NMSCs are Basal Cell Carcinoma (BCC) and Squamous Cell Carcinoma (SCC), occurring at a ratio of about 4∶1 and accounting for about 90% of all skin cancers diagnosed globally [3], [4]. As these cancers are not reported to cancer registries in most countries, precise statistics of NMSC are generally not available. However, it is estimated that between two and three million people are diagnosed worldwide each year, with an average annual increase of 3% to 8% in White populations in Australia, Europe, the United States, and Canada over the last 30 years [5], [6]. The global incidence rates vary by skin complexion and geographical region and are expected to continue to rise in the coming years, due to growing exposure to ultraviolet (UV) sunlight associated with increased sun-seeking behaviors and depletion of stratospheric ozone [7], [8].

Phenotype characteristics, environmental exposures, and genetic predisposition appear to be risk factors for the development and progression of NMSC. Studies on humans and animals suggest that ambient solar radiation, in particular, plays an important role in the pathogenesis of these skin malignancies [9], [10]. Although epidemiological findings show that NMSC occurrence increases with increasing sunlight exposure, and overall estimates from meta-analyses suggest that NMSC is associated with sunlight exposure at the workplace, there is discordance among the results reported by individual occupational studies, particularly for BCC [11]. There is also ongoing controversy regarding the relevance of occupational exposure to artificial UV radiation as a possible skin carcinogen [12]. Other environmental and occupational exposures, for instance to arsenic, polycyclic aromatic hydrocarbons, and ionizing radiation have been linked to NMSC [13]–[16]. Lifestyle factors such as indoor/outdoor tanning-related behaviors, and host characteristics including medical history and familial susceptibility are also associated with an enhanced risk of NMSC [17], [18].

A large number of people are exposed to varying levels of UV radiation at the workplace. However, the number of epidemiological studies focusing on NMSC occurrence in workers is limited and the findings are contradictory. Moreover, most studies are limited by a lack of individual exposure assessment, and are based only on census and registry data, or occupations/industries (e.g., outdoor vs. indoor) as surrogates of exposure. Also, important confounders (e.g., non-occupational UV exposure, other relevant exposures) and/or effect-modifiers (e.g., skin complexion, individual UV sensitivity) were not sufficiently addressed in prior work [19], [20]. The present study investigated the relationship between exposure to occupational UV radiation and NMSC in a large multicenter case-control study conducted in Central and Eastern Europe. For each participant, expert assessment was used to ascertain the lifetime work-related UV exposure from occupational sources. Detailed information on a number of other potential risk factors and effect modifiers was collected and used to adjust or stratify the associations. The main objectives of this study were to establish whether exposures to natural and artificial UV radiation at the workplace are linked to NMSC, and to quantify the associated risks.

## Materials and Methods

### Study Population

The study results are based on data collected during the Arsenic Health Risk and Molecular Epidemiology (ASHRAM) Study, a hospital-based incident case-control study conducted on White residents of three European countries (8 counties located in Hungary, Romania, and Slovakia), between January 2003 and September 2004. All participants provided written informed consent, and the privacy of the study participants and the confidentiality of the information were assured according to the principles of the Helsinki Declaration. The project was reviewed and approved by the Ethical Committees for participating institutions in each contributing country, including: Hungary, the Ethical Committee of the National Health Research Council and the Regional Ethical Committees of the Szentgyörgyi Albert University of Szeged and of the Kecskemét, Gyula, and Szolnok County Hospitals; Romania, the Ethical Committees of the Arad and Bihor County Public Health Departments, and of the Arad and Oradea County Hospitals; Slovakia, the Ethical Committees of the Nitra, Nove Zamky, Levice, and Ziar nad Hronom State Health Institutes, and of the Banska Bystrica, Nitra, Brezno, Nove Zamky, Levice, and Nova Bana Hospitals; and the United States, Institutional Review Board of the University at Albany, State University of New York. The primary aim of the ASHRAM Study was to investigate the carcinogenic role of arsenic exposure via drinking water related to skin, kidney, and bladder cancers.

Skin cancer cases, aged 30–79 years, and having lived in the study area for at least one year, consisted of NMSC newly diagnosed at county hospitals (International Classification of Disease –10^th^ Revision, Codes C44). All cases were confirmed by histological examination or dermatology specialist opinion. Controls were selected from general surgery in-patients (appendicitis, abdominal hernia, duodenal ulcer, cholelithiasis), and from orthopedic or trauma patients (fractures). Controls residing in the study area for at least one year were frequency matched to cases by county of residence, sex, and 5 year age range. A detailed description of participant recruitment has been published previously [21].

### Occupational Exposure Assessment

Participants were interviewed in the hospital or at home within 3 months of enrollment using a questionnaire developed specifically for the ASHRAM Study [21]. Questionnaire items included demographic characteristics, socioeconomic status, medical history, lifestyle factors, such as smoking and solar radiation exposure, and detailed residential and occupational histories. It also contained questions on skin characteristics including complexion and sensitivity to UV radiation.

Exposure to occupational risk factors with potential carcinogenic effects was based on self-reported occupational history. Interviews collected information for each job title held for at least one year over the lifetime, including duration of working, full-time or part-time status, employer, and industry/activity. Additional information was collected on job tasks with potential exposure to relevant hazardous agents. Occupational exposures were ascertained by local experts in industrial hygiene or occupational health, who were blinded to the case status. Job histories for each participant were examined and if necessary, a job was split into several homogeneous periods to reflect temporal changes in technology or tasks.

Job titles and industries were coded according to the International Standard Classification of Occupations [22] and the Classification of Economic Activities in the European Community [23]. Occupational exposures to 27 agents or groups of agents, including natural and artificial UV radiation, were categorized by intensity, frequency, and a confidence factor for each job, using a semi-quantitative, three-point scale job-exposure matrix that was previously developed to study ocular melanoma and adapted for use in our study [24]. The intensity of natural UV exposure was coded as “high” for participants working in outdoor occupations involving fishing, “medium” for agriculture related occupations (e.g., farming, gardening, animal husbandry), forestry, construction, and military service, and “low” for other outdoor occupations. The intensity of artificial UV exposure was coded as “high” for participants with indoor occupations such as arc welding, “medium” for metal smelting, and “low” for other jobs involving machinery repair and fabricated metal products manufacture. The frequency of exposure for each job period was estimated as the percentage of a 40-hour work week during which exposure occurred. Frequency was coded as “high” for UV exposure more than 2.5 hours a day, “medium” for UV exposure from 0.5 to 2.5 hours a day, and “low” for participants with UV exposure from 5 min to 0.5 hours a day. The confidence factor represents the degree of certainty that the worker has been exposed to UV radiation and was coded as “high” for certain exposure, “medium” for probable exposure and “low” for possible exposure.

### Statistical Analysis

Occupational exposure to natural and artificial UV radiation was considered using two indices: “ever” exposure and cumulative exposure over the lifetime. A subject was classified as ever exposed if any of the reported jobs involved UV radiation from the sun (e.g., outdoor occupations), or from artificial sources (e.g., welding work). If none of the jobs over a lifetime was associated with UV radiation, the subject was classified as “never” exposed. The cumulative lifetime exposure (CLE) was calculated by summing over a participant’s working lifetime the products of exposure semi-quantitative scores and the exposure duration for each job period, as presented in the following equation:
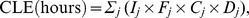
where, *I_j_* is the intensity of exposure for the *j*th job (*I* = 0.25 (low), 0.50 (medium), 1.00 (high)), *F_j_* is the frequency of exposure for the *j*th job (*F* = 0.03 (low), 0.18 (medium), 0.65 (high)), *C_j_* is the confidence factor of exposure assigned to the *j*th job (*C* = 0.25 (low), 0.50 (medium), 1.00 (high)), and *D_j_* is the duration of exposure in hours (*D* = 2,000 hours per working year).

The continuous cumulative exposure variable was further categorized based on tertiles of the distribution among controls with participants never exposed to workplace UV radiation defined as the reference category. Frequency distributions of exposures and demographic characteristics were characterized and compared by case status using chi-square tests.

Odds ratios (OR) and 95% confidence intervals (95% CI) were used to estimate associations between occupational exposure to UV radiation and NMSC using unconditional logistic regression. Based on the literature, a number of factors possibly associated with the risk of NMSC development and the likelihood of working in agricultural or industrial settings with UV exposure were considered, including skin complexion, propensity for sunburn, cancer history, education as a proxy for socio-economic status, tobacco smoking, recreational UV exposure, arsenic exposure at work, and arsenic exposure through consumption of contaminated drinking water. Skin complexion, family history of cancer, and lifetime average exposure to arsenic in drinking water were identified as confounding factors (i.e., statistically significant association with both occupational exposure to UV radiation in controls and NMSC among unexposed participants) and were therefore included in the final multivariable regression models, along with the matching variables sex, age, and county of residence.

Skin complexion is a critical modifier for the effect of UV radiation exposure on NMSC. We included skin complexion (i.e., light vs. medium/dark) evaluated in multivariable regression models by the inclusion of two-way interaction terms between skin complexion and UV exposure. Effect modification by skin cancer histology and anatomical location was also considered by stratified analyses.

To account for the reported latency [25] between UV radiation exposure at work and the development of skin cancer, association estimates for NMSC were also calculated for 20-, 25- and 30-year lag periods. In these analyses, the lag periods prior to study participation were considered to be unexposed. All analyses were conducted using SAS 9.2 statistical software (SAS Institute, Cary, NC, USA). Statistical significance was defined as p<0.05 for main effects and p<0.10 for interaction terms, using two-tailed tests.

## Results

The response rate for the ASHRAM study was 81.6% for cases and 90% for controls. A total of 618 NMSC cases and 527 controls were included in the present study (Table 1). Cases tended to be older and to have fewer years of education compared to the control group. Cases also had a higher tendency for light skin complexion, propensity for sunburn, a family history of cancer, more lifetime hours of recreational sun exposure, and arsenic exposure at work. Unadjusted prevalence estimates for smoking and exposure to moderate or high lifetime average concentration of arsenic in drinking water were higher in the control group than in cases.

**Table 1:** Selected characteristics of controls and cases of non-melanoma skin cancer (NMSC).

Characteristic	Controls		NMSC		p-value^b^
	n^a^	%	n^a^	%	
**Sex**					0.06
Female	255	48.4	333	53.9	
Male	272	51.6	285	46.1	
**Age (years)** c					<0.001
<52	136	25.8	78	12.6	
53–61	131	24.9	119	19.3	
62–70	144	27.3	183	29.6	
≥71	116	22.0	238	38.5	
**Country**					<0.001
Hungary	240	45.5	170	27.5	
Romania	156	29.6	218	35.3	
Slovakia	131	24.9	230	37.2	
**Number of years of education** c					0.006
≥13	114	21.8	144	23.4	
11–12	143	27.3	115	18.7	
9–10	53	10.1	64	10.4	
<8	214	40.8	293	47.6	
**Smoking**					<0.001
Never smoked	276	52.5	392	63.5	
Past smoker	143	27.2	156	25.3	
Current smoker	107	20.3	69	11.2	
**Family history of cancer**					<0.001
No	412	78.2	418	67.6	
Yes	115	21.8	200	32.4	
**Skin complexion**					<0.001
Medium/dark	310	58.9	312	50.6	
Light	216	41.1	305	49.4	
**Propensity for sunburn**					0.004
No change/tan without sunburn	226	43.6	206	33.8	
Mild sunburn that becomes a tan	156	30.1	191	31.4	
Sunburn without blisters	79	15.2	126	20.7	
Sunburn with blisters	58	11.2	86	14.1	
**Lifetime cumulative exposure to sun on weekend days (hours)** c					<0.001
Very low (<1,589)	130	25.1	107	17.4	
Low (1,589–2,390)	130	25.1	129	21.0	
Moderate (2,390–3,564)	130	25.1	183	29.8	
High (>3,564)	129	24.9	195	31.8	
**Arsenic exposure at work**					<0.001
Never	445	84.4	471	76.2	
Ever	82	15.6	147	23.8	
**Lifetime average concentration of arsenic in drinking water (µg/L)** c					0.005
Very low (<0.70)	147	28.1	212	34.6	
Low (0.70–1.81)	114	21.8	151	24.7	
Moderate (1.82–16.65)	132	25.2	108	17.7	
High (>16.65)	130	24.9	141	23.0	

a Total number of participants varies due to missing data for some covariates;

b Chi-square test for differences between case and control group calculated using unmatched data;

c Quartiles of the control group distribution.

A total of 5,589 job periods (4.9 job periods per subject on average), were coded based on job title and employer activity as reported during the interview. Workplace exposure to natural (i.e., sunlight) UV radiation was ascertained for 511 job periods and to artificial UV radiation for 249 job periods. Out of 227 participants who were classified as ever being exposed to occupational UV radiation, 135 were exposed to sunlight only, 69 to artificial UV radiation only, and the remaining 23 had been exposed to both natural and artificial UV radiation. The small number of participants exposed to both natural and artificial UV light did not allow for a separate analysis. As anticipated, a high proportion of participants ever exposed to natural UV radiation were involved in agriculture (23%), military service (19%), construction (10.5%), transport (9.5%), and forestry (6%). The participants ever exposed to artificial UV radiation were predominantly workers in machinery manufacturing (39%), blacksmiths, toolmakers, and machinery fitters/assemblers (17%), and plumbers, welders, and sheet metal workers (10%).

The lifetime prevalence of ever exposure to natural UV radiation was 13.8% for cases (N = 78) and 11.9% for controls (N = 57), while the prevalence of exposure to artificial UV radiation was only 6.6% for cases (N = 34) and 7.7% for controls (N = 35). Table 2 shows the multivariable adjusted odds ratios for NMSC associated with occupational UV radiation (i.e., from any sources (including 9 controls (1.7%) and 14 cases (2.3%) with both sources of exposure), from natural sources only, and from artificial sources only) for ever exposure vs. never exposure, as well as for the cumulative index of exposure (tertiles), with and without a 30-year lag period. Significantly lower adjusted odds ratios of NMSC were observed for ever exposure to occupational natural UV radiation compared to never exposure (OR 0.47, 95% CI 0.27–0.80), and for lifetime cumulative exposure in the lower (OR 0.43, 95% CI 0.19–0.94) and medium (OR 0.34, 95% CI 0.15–0.73) tertiles, compared to the never exposed group. The multivariable logistic regression estimates, adjusted for potential confounders, showed no association between NMSC and workplace exposure to artificial UV radiation, with odds ratios ranging from 1.73 (95% CI 0.76–3.93) for lifetime cumulative exposure in the lower tertile to association estimates below the null for the medium and higher tertiles. The latency analysis also showed a similar pattern, although the number of exposed participants decreased and the association estimates were less precise. Therefore, the study findings including lag periods are not reported for the subsequent analyses.

**Table 2:** Adjusted odds ratios between occupational exposure to ultraviolet radiation (UVR) and non-melanoma skin cancer (NMSC).

	No lag				30-year lag			
Occupational exposure index	Controls	NMSC			Controls	NMSC		
	n^a^	n^a^	OR^b^	95% CI	n^a^	n^a^	OR^b^	95% CI
**Never exposed**	421	485	1.00	(referent)	448	499	1.00	(referent)
**Ever exposed**								
Any UVR^c^	101	126	0.72	0.48–1.08	74	112	0.82	0.54–1.24
Natural UVR	57	78	0.47	0.27–0.80	41	76	0.65	0.38–1.12
Artificial UVR	35	34	1.17	0.67–2.05	26	29	1.20	0.66–2.21
**Cumulative lifetime exposure**								
Any UVR^c^								
Tertile 1 (≤875 hours)	34	37	0.85	0.48–1.52	36	44	0.89	0.52–1.54
Tertile 2 (875.5–3237.5 hours)	34	27	0.50	0.27–0.93	16	25	0.72	0.35–1.47
Tertile 3 (>3237.5 hours)	33	62	0.83	0.47–1.45	22	43	0.78	0.42–1.45
Natural UVR								
Tertile 1 (≤1225 hours)	20	20	0.43	0.19–0.94	23	27	0.42	0.21–0.86
Tertile 2 (1225.5–5075 hours)	19	19	0.34	0.15–0.73	5	22	1.45	0.50–4.17
Tertile 3 (>5075 hours)	18	39	0.66	0.32–1.34	13	27	0.70	0.32–1.50
Artificial UVR								
Tertile 1 (≤570 hours)	12	18	1.73	0.76–3.93	15	19	1.61	0.75–3.45
Tertile 2 (570.5–2326.5 hours)	12	7	0.82	0.29–2.34	7	6	0.70	0.22–2.20
Tertile 3 (>2326.5 hours)	11	9	0.90	0.33–2.44	4	4	0.80	0.16–3.99

a Total number of participants varies due to missing data for some covariates;

b Adjusted odds ratios (95% CI) for sex, age, county of residence, family history of cancer, skin complexion, and lifetime average arsenic concentration in drinking water;

c Any UVR consists of natural UV, artificial UV, and both (results omitted due to scarce data); associations were estimated in two separate multivariable logistic regression models.

Table 3 reports the findings for occupational ever exposure to UV radiation and NMSC stratified by anatomical site. The odds ratios for NMSC were significantly lower in participants ever exposed to natural UV at the workplace, for sites often exposed to the sun such as face, head, and neck (OR 0.47, 95% CI 0.27–0.83) as well as for sites less frequently exposed to sunlight such as the trunk and the upper and lower extremities (OR 0.46, 95% CI 0.22–0.99). There was no apparent association between workplace exposure to artificial UV radiation and NMSC for any anatomical site investigated.

**Table 3:** Adjusted odds ratios between occupational exposure to ultraviolet radiation (UVR) and non-melanoma skin cancer (NMSC) stratified by anatomical site.

Occupational exposure index	Controls	NMSC					
		Face, head, and neck			Other sites		
	n^a^	n^a^	OR^b^	95% CI	n^a^	OR^b^	95% CI
**Never exposed**	421	248	1.00	(referent)	81	1.00	(referent)
**Ever exposed**							
Any UVR^c^	101	86	0.75	0.47–1.19	29	0.59	0.30–1.13
Natural UVR	57	54	0.47	0.27–0.83	20	0.46	0.22–0.99
Artificial UVR	35	20	1.51	0.77–2.94	7	1.07	0.41–2.80

a Total number of participants varies due to missing data for some covariates;

b Adjusted odds ratios (95% CI) for sex, age, county of residence, family history of cancer, skin complexion, and lifetime average arsenic concentration in drinking water;

c Any UVR consists of natural UV, artificial UV, and both (results omitted due to scarce data); associations were estimated in two separate multivariable logistic regression models.

Table 4 describes the NMSC results for statistical interactions to assess effect modification of UV exposure-NMSC associations by skin complexion. There was evidence of effect modification by skin complexion for UV radiation exposure from the sun, as demonstrated by significant statistical interactions for ever exposure, with significant decreases in the adjusted odds ratio of NMSC (OR 0.32, 95% CI 0.16–0.61) only among participants reporting a light skin complexion. Similar results were found for medium lifetime cumulative exposure (OR 0.15, 95% CI 0.06–0.41). There were an insufficient number of participants to permit estimates of effect modification for associations between tertiles of artificial UV cumulative exposure.

**Table 4:** Modifying effect of skin complexion on adjusted odds ratios between occupational exposure to ultraviolet radiation (UVR) and non-melanoma skin cancer (NMSC).

Occupational exposure index and skin complexion	Controls	NMSC		
	n^a^	n^a^	OR^b^	95% CI
**Ever exposed**				
Any UVR* **^c^**				
Medium/dark skin	59	77	0.93	0.57–1.52
Light skin	42	49	0.49	0.28–0.84
Natural UVR*				
Medium/dark skin	30	44	0.65	0.34–1.26
Light skin	27	34	0.32	0.16–0.61
Artificial UVR				
Medium/dark skin	23	22	1.17	0.59–2.30
Light skin	12	12	1.16	0.45–2.96
**Cumulative lifetime exposure** d				
Any UVR* **^c^**				
Tertile 1 (≤875 hours)				
Medium/dark skin	24	22	0.84	0.41–1.69
Light skin	10	15	0.70	0.28–1.78
Tertile 2 (875.5–3237.5 hours)				
Medium/dark skin	13	18	1.02	0.46–2.29
Light skin	21	9	0.20	0.08–0.51
Tertile 3 (>3237.5 hours)				
Medium/dark skin	22	37	0.92	0.47–1.81
Light skin	11	25	0.77	0.34–1.76
Natural UVR*				
Tertile 1 (≤1225 hours)				
Medium/dark skin	13	11	0.41	0.16–1.10
Light skin	7	9	0.33	0.11–1.02
Tertile 2 (1225.5–5075 hours)				
Medium/dark skin	5	11	1.06	0.32–3.50
Light skin	14	8	0.15	0.06–0.41
Tertile 3 (>5075 hours)				
Medium/dark skin	12	22	0.71	0.30–1.68
Light skin	6	17	0.63	0.22–1.77

a Total number of participants varies due to missing data for some covariates;

b Adjusted odds ratios (95% CI) for sex, age, county of residence, family history of cancer, skin complexion, and lifetime average arsenic concentration in drinking water;

c Any UVR consists of natural UV, artificial UV, and both (results omitted due to scarce data); associations were estimated in two separate multivariable logistic regression models;

d Cumulative lifetime exposure is not showed for artificial UVR because of small numbers per strata;

* Significance of the Wald Chi-Square test for interaction at p<0.10.

We conducted an additional subgroup analysis of the BCC histologic type (Table 5). The results were similar to those for total NMSC. A significantly reduced adjusted odds ratio of BCC (OR 0.43, 95% CI 0.25–0.74) was detected in association with natural UV radiation exposure. The analysis of BCC risk modification by skin complexion also identified significantly lower odds ratios, but only among participants with light skin complexion ever exposed or exposed to medium cumulative levels of natural UV radiation at work.

**Table 5:** Adjusted odds ratios between occupational exposure to ultraviolet radiation (UVR) and basal cell carcinoma (BCC), and modifying effects by skin complexion.

Occupational exposure index and skin complexion	Controls	BCC		
	n^a^	n^a^	OR^b^	95% CI
**Ever exposed**				
Any UVR* **^c^**	101	102	0.64	0.42–0.99
Medium/dark skin	59	65	0.91	0.54–1.52
Light skin	42	37	0.39	0.22–0.70
Natural UVR*	57	63	0.43	0.25–0.74
Medium/dark skin	30	38	0.67	0.35–1.32
Light skin	27	25	0.26	0.13–0.52
Artificial UVR	35	27	1.06	0.59–1.93
Medium/dark skin	23	18	1.16	0.57–2.38
Light skin	12	9	0.91	0.33–2.45
**Cumulative lifetime exposure** d				
Any UVR* **^c^**				
Tertile 1 (≤875 hours)	34	31	0.74	0.40–1.34
Medium/dark skin	24	19	0.83	0.39–1.74
Light skin	10	12	0.64	0.24–1.67
Tertile 2 (875.5–3237.5 hours)	34	21	0.41	0.21–0.78
Medium/dark skin	13	13	0.88	0.37–2.11
Light skin	21	8	0.19	0.07–0.48
Tertile 3 (>3237.5 hours)	33	50	0.77	0.43–1.36
Medium/dark skin	22	33	0.97	0.49–1.92
Light skin	11	17	0.53	0.22–1.26
Natural UVR*				
Tertile 1 (≤1225 hours)	20	16	0.36	0.16–0.80
Medium/dark skin	13	9	0.40	0.14–1.10
Light skin	7	7	0.31	0.10–1.02
Tertile 2 (1225.5–5075 hours)	19	16	0.32	0.14–0.70
Medium/dark skin	5	10	1.11	0.33–3.69
Light skin	14	6	0.12	0.04–0.35
Tertile 3 (>5075 hours)	18	31	0.62	0.30–1.28
Medium/dark skin	12	19	0.74	0.31–1.78
Light skin	6	12	0.47	0.16–1.39

a Total number of participants varies due to missing data for some covariates;

b Adjusted odds ratios (95% CI) for sex, age, county of residence, family history of cancer, skin complexion, and lifetime average arsenic concentration in drinking water;

c Any UVR consists of natural UV, artificial UV, and both (results omitted due to scarce data); associations were estimated in two separate multivariable logistic regression models;

d Cumulative lifetime exposure is not showed for artificial UVR because of small numbers per strata;

* Significance of the Wald Chi-Square test for interaction at p<0.10.

## Discussion

The present case-control study of more than 1,100 participants investigated NMSC in relation to natural and artificial UV radiation exposure in agricultural and industrial workplaces, in three Central and Eastern European countries. The study results suggested a weak inverse association of NMSC, mainly due to BCC, with workplace exposure to natural UV radiation, and no significant relationship with artificial UV radiation. The inverse association was limited to participants with light skin complexion.

UV radiation exposure does not increase human health risks monotonically, but rather demonstrates a hormesis dose-response relationship due to biological determinants such as vitamin D levels and behavioral factors including UV exposure pattern, and amount and type of radiation. Minimum risks for adverse health effects occurs at an optimal exposure, according to skin complexion and individual UV sensitivity, while increased disease risk is observed with very low level UV or very high levels [26]–[28]. The carcinogenetic role of UV radiation in NMSC has been investigated extensively, solar radiation being classified by the International Agency for Research on Cancer (IARC) as a Class 1, “definite” human carcinogen [9]. The underlying physiological mechanisms of carcinogenesis involve direct DNA damage, as well as alterations in DNA repair and immune response pathways. On the other hand, moderate UV exposure is essential for the production and preservation of adequate vitamin D levels, which has itself been suggested to reduce the risk of cancer. Several environmental [29]–[31] and occupational [32]–[34] epidemiological studies have shown associations between sunlight exposure to UV radiation and a reduced risk for various cancers including colorectal, breast, prostate, kidney, melanoma, and non-Hodgkin lymphoma. Vitamin D immune-modulatory mechanisms and regulatory effects on the cell cycle have been proposed as mechanisms driving these earlier results [27], [35].

Given the substantial volume of literature corroborating solar radiation exposure as an important risk factor for skin cancer, the present findings of weak inverse associations between sun exposure at work and BCC seem contradictory. Yet, comparable results were reported in several other epidemiologic studies of UV-induced BCC at the workplace published between 1995 and 2006, and recently reviewed by Bauer et al. [36]. This meta-analysis concluded the existence of a positive association, but individual study results were discordant: seven of those studies showed non-significant inverse associations or no effect, six studies reported positive but not significant associations and 11 studies reported significant positive associations between occupational UV exposure and the risk of BCC. Another recent review also concluded that there is no consistent evidence of a relation between sun exposure at work and BCC [11]. Two nationwide studies conducted in Denmark and Finland, published in 1999–2010, reported significantly reduced risks of NMSC and BCC related to outdoor occupations in construction, agriculture, farming, forestry, and fishing [37], [38]. The present study findings for workplace exposure to artificial UV radiation and the risk of NMSC agree with those previous epidemiologic results, which indicate that to date, there is no evidence of an increased skin cancer risk associated with artificial UV exposure at work.

The lack of an increased NMSC risk from occupational exposure to natural UV radiation, and significant protective effects against NMSC among participants with a light skin complexion, may be linked to a modification of behaviors towards adopting personal sun protection measures. Although sun protection behaviors vary considerably by occupation, sex, age, education, and local sun-related habits, a number of studies reported that outdoor workers and individuals with sun sensitive skin types are more likely to employ sun safety practices such as wearing a hat or protective clothes [39]–[41]. Furthermore, the weight of evidence suggests a higher risk of BCC in relation to intermittent intense sun exposure, and recreational sun exposure early in life compared to chronic, and occupational exposures [18], [42]. Chronic exposure such as that in the workplace appears to be more closely related to SCC risk. Several studies found a relationship between SCC development and long-term workplace cumulative exposure to sun radiation [19], [43]. However, the number of exposed subjects diagnosed with SCC was too small in the present study to support a subgroup analysis of this histologic type (i.e., 9 subjects exposed to natural UV, 6 subjects exposed to artificial UV radiation).

Investigators previously reported large spatial differences in the NMSC risk among White populations, with reported incidences being about 5-fold and 7-fold higher in the U.S. than in Europe, and about 50-fold and 100-fold higher in Australia compared to Europe [44]. Proximity to the equator is known to be a strong predictor of skin cancer risk (i.e., decline of NMSC rates with increasing latitude due to lower ambient UV radiation), and thus the Central European location of this study may explain in part the findings [1]. A recent meta-analysis of studies on BCC occurrence in relation to occupational sun exposure confirmed the strong inverse association between geographical latitude and the risk of BCC [36].

The present study has several limitations. First, the use of hospital controls may be of concern when the source population from which cases originate is not adequately represented. Various strategies were undertaken to minimize the potential for this type of selection bias and they were extensively discussed in a previous publication [21]. Secondly, due to the historical exposure assessment, misclassification of exposure is of concern and may change the association estimates. Misclassification bias can be introduced in the study during data collection (e.g., recall bias, interviewer bias) or during the exposure reconstruction process. Differential recall of occupational histories by case status (i.e., in which controls are more likely to omit or incorrectly report a job title than cases) might lead to an overestimate of UV effects. However, if the degree of misclassification is similar in cases and controls, then the odds ratios are likely to be biased towards the null hypothesis of no association. Several previous studies compared the accuracy and completeness of occupational reporting between cases and controls and found small variations [45], [46]. In the current study, recall bias was minimized by using a structured questionnaire that was piloted in the study area and administered face-to-face. To reduce potential bias on the part of the interviewers, interviews were conducted according to a written protocol by investigators who participated in training workshops. Neither participants nor interviewers were made aware of the current occupational UV exposure study hypothesis.

Occupational exposure to UV radiation was not self-reported, but was reconstructed by subject-matter experts using job histories. Study exposures were limited to jobs of at least one year duration; we did not capture short-term work such as summer seasonal agricultural jobs, or migration associated outdoor employment because these practices are uncommon in the communities studied. The experts, who were blinded to the disease status, assigned the exposure based on an occupational coding manual. The experts participated in several training workshops and validation exercises. As a result, any assigned exposure misclassification is expected to be similar for cases and controls and thus, will cause an underestimation of UV effects. Furthermore, it is unlikely that any systematic bias in the assignment of exposure by experts would be limited to only light-skinned participants, the group in which a decreased risk is detected, further suggesting that any exposure misclassification is likely to have been non-differential. Analysis of exposure patterns among the subset of participants with estimated high intensity UV radiation exposure would have the advantage of reducing potential exposure misclassification bias as these are important in the etiology of NMSC. However, the small number of participants with high intensity UV radiation exposure at work (i.e., 7 participants for natural UV, 1 participant for artificial UV) precluded a subgroup analysis.

This study has a number of strengths compared to studies focusing on the association between NMSC and occupational exposure to UV radiation. The large sample size and the pathological verification of 94% of the NMSC cases facilitated a subgroup analysis by histological type and by anatomical site. While this study had sufficient statistical power to detect relatively small associations, the number of cases diagnosed with tumors located on body sites usually not exposed to solar radiation was rather small. Other methodological strengths of this study include the short period of time for recruitment cases and controls (21 months), the use of incident cases, and the high participant response rate (>85%). Furthermore, the associations were adjusted for important confounding factors and were reported by skin complexion.

### Conclusions

The study results do not provide support for an increased risk of NMSC in association with workplace exposure to natural or artificial UV radiation. These findings are consistent with weak, null or inverse associations previously reported in epidemiologic studies, particularly for BCC. Our results might be attributed to the low level of UV exposure among participants, and to exposure misclassification. The protective effect we observed among participants with light skin complexion suggests that they are using adequate personal sun-protection measures. These results add to the evidence that moderate sunlight exposure might decrease the risk of some types of cancer, likely in association with sun-protection behaviors. Further investigation focusing on individual genetic susceptibility and potential interactions with other exposures at work and with low-level environmental exposures will be conducted in order to achieve a more complete knowledge of the etiology and effective prevention methods for human skin malignancies.
